# Applying text-mining to clinical notes: the identification of patient characteristics from electronic health records (EHRs)

**DOI:** 10.1186/s12911-025-03137-x

**Published:** 2025-08-12

**Authors:** Simone ten Hoope, Koen Welvaars, Kylian van Geijtenbeek, Mellanie Klok-Everaars, Sander van Schaik, Fatma Karapinar-Çarkit

**Affiliations:** 1 Department of Clinical Pharmacy, OLVG Hospital, Amsterdam, The Netherlands; 2 Department of Data Science, OLVG Hospital, Amsterdam, The Netherlands; 3 Department of Neurology, OLVG Hospital, Amsterdam, The Netherlands; 4 Department of Clinical Pharmacy & Toxicology, Maastricht University Medical Center+, Maastricht, The Netherlands; 5 Department of Clinical Pharmacy, Cardiovascular Research Institute Maastricht, CARIM, Maastricht University, Maastricht, The Netherlands

**Keywords:** Natural language processing (NLP), Rule-based (RB) algorithms, Patient characteristics, Text-mining, Electronic health records (EHRs)

## Abstract

**Background:**

Clinical notes contain information on critical patient characteristics, which, if overlooked, could escalate the risk of adverse events as well as miscommunication between the healthcare professional and the patient. This study investigates the feasibility of employing text-mining to extract patient characteristics from Electronic Health Records (EHRs) and compares the effectiveness of text-mining against human intelligence for identifying four patient characteristics: language barrier, living alone, cognitive frailty and non-adherence.

**Methods:**

A manual “golden” standard was created from 1,120 patient files (878 patients) that had unplanned hospital readmissions. Each patient was categorized in one (or multiple) of the four characteristics with supporting clinical notes extracted from their EHRs. For simple terminology, a rule-based (RB) SQL query was used, and for complex terms, Named Entity Recognition (NER) models were used. Model performance was compared to the manual standard. The primary outcomes were recall, specificity, precision, negative predictive value (NPV) and F1-score.

**Results:**

Performance of each patient characteristic was evaluated using a separate train/test dataset. An additional validation dataset was used for the NER models. Within the train/test set, the language barrier RB query achieved a recall of 0.99 (specificity of 0.96). The living alone NER model achieved a recall of 0.86 (specificity of 0.94) on the train/test set and a recall of 0.81 (specificity of 1.00) on the validation set. In that same order, the cognitive frailty model yielded a recall of 0.59 (specificity 0.76) on the train/test set and a recall of 0.73 (specificity 0.96) on the validation set. The NER model for non-adherence achieved a recall of 0.75 (specificity of 0.99) on the train/test set, and a recall of 0.90 (specificity of 0.99) on the validation set. The models showed the tendency to overestimate the presence of patient characteristics such as identifying a family member’s language barrier as the patient’s.

**Conclusion:**

This study successfully demonstrated the feasibility of applying text-mining to identify patient characteristics from EHRs. Also, it seems for more complex terminology, NER models outperform the rule-based option. Future work involves refining these models for broader application in clinical settings.

**Clinical trial number:**

Not applicable.

**Supplementary Information:**

The online version contains supplementary material available at 10.1186/s12911-025-03137-x.

## Introduction

Electronic health records (EHRs) are a digital collection of patient information and are pivotal in both patient care and medical research [1–3]. Information in EHRs comprise two categories of data: structured and unstructured [4]. Structured data includes quantifiable variables such as patient demographics, diagnostic codes and laboratory results, while unstructured data, predominantly textual, refers to clinical notes and discharge records composed by healthcare professionals (excluding attachments) [1, 5]. Notably, clinical notes encapsulate critical patient characteristics, such as language barriers, forgetfulness, non-adherence or solitary living conditions, which, if overlooked, could escalate the risk of adverse events as well as miscommunication between the healthcare professional and the patient [6–8]. Despite their value, clinical notes are frequently underutilized in analyses, largely due to challenges in interpreting these notes, which often contain grammatical errors, abbreviations, and diverse expressions specific to individual healthcare professionals [4, 5, 9]. Furthermore, with 80% of EHRs containing clinical notes with unstructured texts, extracting information on patient characteristics is difficult [9]. 

The creation of a system that converts clinical notes into structured data can make these patient characteristics readily accessible to healthcare professionals, thereby facilitating personalized care. Text-mining, a branch of artificial intelligence, focuses on converting free, unstructured textual data into standardized, structured data. Two primary text-mining techniques are often used: SQL Rule-based (RB) queries and Natural Language Processing (NLP) token classification, specifically Named Entity Recognition (NER) [2]. 

RB queries are predefined text rules, making them suitable for capturing clear-cut terms and offering straightforward interpretation [10]. However, RB queries fall short in situations with significant descriptive variability, spelling errors, or absence of precise terms, often leading to rule expansion with the proliferation of conflicting rules, potentially resulting in misclassifications of the patients [3, 11]. Conversely, NER models, using token classification, are adept at processing text even with spelling inconsistencies, effectively managing terms with variations [10, 12]. Nevertheless, NER models employ deep learning, creating complex ‘black box’ neural networks that are challenging to understand [13]. 

Prior research has demonstrated the efficacy of text-mining, including identifying adverse drug reactions [14, 15] and disease symptoms from EHRs [12], and applying them for diagnosing disorders and identifying patients with a fall risk [3, 16–19]. Text-mining has also demonstrated efficacy in identifying and extracting social risk factors that affect access to care and health outcomes from clinical notes [20, 21]. To the best of our knowledge, few studies applied a specific text-mining approach to identify certain patient characteristics - language barrier, living alone, cognitive frailty and non-adherence – that are known risk factors for medication-related hospital readmissions, from unstructured clinical notes in EHRs [8, 22]. 

This research primarily aims to develop a method for applying text-mining to identify patient characteristics from EHRs. The secondary aim is to evaluate the performance of text-mining against human intelligence measured in recall, specificity, precision, negative predictive value (NPV) and the F1-score.

## Method

### Design and setting

This observational study was conducted at OLVG, a teaching hospital in Amsterdam, the Netherlands, from February to July 2022. This study protocol received a waiver from the local ethics committee with registration number 16–028 (Advies Commissie Wetenschappelijk Onderzoek-Medisch Ethische Commissie, ACWO-MEC, OLVG Hospital, Amsterdam) as it was outside the scope of the Medical Research Involving Human Subjects Act (Wet Medisch-wetenschappelijk Onderzoek, WMO) (MEC-2016-346). Patient data were obtained and handled in accordance with applicable privacy regulations.

### Study population

Participants were included based on a prior study involving patients with unplanned hospital readmissions from July 2016 to February 2018 [8, 22]. Adult patients who experienced unplanned readmissions within 30 days of discharge from high-readmission departments (surgery, cardiology, pulmonology, neurology, internal medicine, gastroenterology, psychiatry) were included. Exclusions were transfers to other hospitals during initial hospitalization and if the readmission was unrelated to the initial admission. This study comprised of 878 patients with 1,120 readmission files. Further detailed information on the initial study procedures can be found in previous studies [8, 22]. 

### Case study

The existing database from the previous study categorized patients according to four patient characteristics: language barrier, living alone, cognitive frailty and non-adherence. Patient characteristics were initially identified using the EHR’s search function (EPIC systems corporation, Verona, Wisconsin, United States) and, if necessary, through manual review of clinical notes. A medical doctor crosschecked a subset of this data. For this study, the existing dataset was re-reviewed to ensure proper classification according to the definitions as stated below. Two researchers examined the EHRs and reclassified the patients where necessary, extracting the supporting clinical notes for each classification (joint review). To account for temporal characteristics, the most recent clinical progress notes were used. Questions about classifications were subsequently reviewed and discussed within the research team, including a senior clinical pharmacist and neurologist to ensure consistency and accuracy. This consensus-based approach was used to construct the human intelligence ‘golden standard’.

### Patient characteristic definitions and classification

The following patient characteristics were extracted:

- *Language barrier*: Defined as communication difficulties between healthcare professionals and patients due to language differences.
- *Living alone*: Defined as residing in a single-person household.
- *Cognitive frailty*: Characterized by symptoms of forgetfulness, dementia or other cognitive issues, with input from geriatricians and neurologists for correct search term selection.
- *Non-adherence*: Described as patients not following prescribed medication regimes or suspected non-compliance.

For language barrier, cognitive frailty and non-adherence, patients were categorized as ‘yes’ (evidence present), ‘no’ (no evidence), or ‘conflicting information’ (inconsistent EHR notes). The category conflicting information was applied only when the research team (including both researchers and hospital pharmacists) agreed that there was ambiguity in the classification based on documentation in EHR. This typically occurred when information from different healthcare professionals was inconsistent or contradictory. For example, a case was classified as conflicting when a physician documented a language barrier, while a nurse noted that the patient could understand the language when spoken slowly. For living situation, patients were classified as living alone, living with others (e.g. a partner, spouse), in an institution (e.g., a nursing home, psychiatric facility), or as conflicting information. This variable is intended to capture whether a patient is truly living alone, as an indicator of social isolation or lack of self-sufficiency.

### Text-mining

**Fig. 1:**
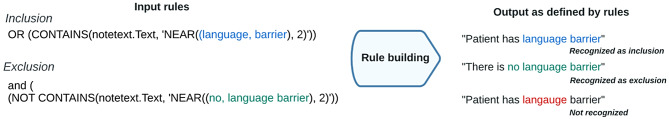
Representation of the RB query. The colored words represent what the query can identify in the EHR. The query only identifies words defined by the creator. Hence, spellingsmistakes are not identified unless specifically stated in a rule. The word “NEAR” in combination with the number defined at the end of the rule (in this case “2”) restricts the words in the rule to a maximum distance of two words apart

Two text-mining techniques were employed: SQL RB queries and NER models (part of NLP token classification). The first technique was an RB SQL query. This structured search statement uses a set of predefined conditions or rules to retrieve data from a database, and is shown in Fig. 1. RB queries can be applied to capture unambiguous terms [10]. 

The descriptions as well as the word distance, collected from the manual standard, were used as input to build the rules based on inclusion and exclusion criteria. The RB query was tested on the entire dataset in SQL Server Management Studio v18.7 (SSMS). In SQL, the RB query had access to the complete EHRs of the included patients (1,120 files). After each execution of the query, results were compared to the manually created ‘golden standard’. An analysis was performed on the first 35 discrepancies between the RB query results and the manual classifications. Based on these discrepancies, the query rules were iteratively adjusted through expert review. To avoid an excessive long list of rules, and potentially conflicting rules, the refinement process was limited to a maximum of five iterations. Adjustments were based on logical consistency, domain expertise, and clinical relevance as determined by the research team.

**Fig. 2:**
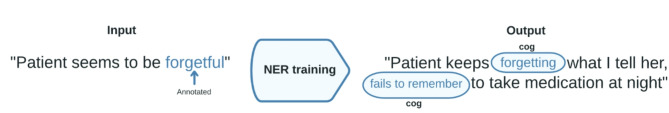
Representation of NER data training. Two persons manually annotated the input based on the annotation list

The second model used was the NER model that identifies and categorizes entities such as names, dates and locations in unstructured text based on an annotation list. Figure 2 represents the training phase, the output “cog” (entity name for cognitive frailty) is what the model learned to define as cognitive frailty. As the obtained data collected was in Dutch, and no NER models were yet available in Dutch, we created our own.

The NER models were trained using clinical notes, with historical indicators (e.g. patient history) excluded. A Dutch annotation list was developed to annotate clinical text sentences, using a SpaCy Python NLP library-compatible annotation tool [23]. Initially, sentences containing the variable were extracted from a training dataset comprising 400 patient files, about 35% of the total dataset, which were randomly selected. No stratification for specific patient characteristics was applied, and all clinical notes were included in annotation.

This subset was thoroughly analyzed to define common terminology describing the characteristics, which were then used to create the annotation list. This list served as the basis for annotating data from our dataset, and served as the foundation for training the NER models. The NER models were implemented using SpaCy v3.2, with the base Dutch configuration optimized for efficiency on CPU (available at spaCy’s official config. repository). The pipeline included the default ‘NER’ component, which is built on a transition-based neural network architecture. No pre-trained embeddings were used beyond those provided in SpaCy’s base configuration. The default training configuration was used, including hyperparameters such as a dropout rate of 0.2, a batch size of 128, and training for up to 30 epochs. Training was performed using CPU resources, in Python v3.8 within Jupyter Notebooks. The final model performance was evaluated using entity-level precision, recall, and F1-score. The annotated codes were saved as a.JSON file, formatted into a DocBin format and loaded into a custom SpaCy pipeline to train the NER neural network. This process was performed using Python v3.8 with Jupyter Notebooks.

### Independent internal validation set

To thoroughly evaluate performance after training, all three developed NER models were additionally evaluated using separate and withheld (internal validation) datasets. A SQL query was used to extract the validation data, and a date filter was applied to extract different notes used in the training data. Therefore ensuring there was no information leakage in the validation set. The living alone NER model was validated using 800 clinical notes, the cognitive frailty model using 1,000 clinical notes, and the non-adherence model using 783 clinical notes. The recall, specificity, precision, negative predictive value (NPV) and the F1-score were also defined for this internal validation set.

### Outcomes and analysis

The primary outcome was creating a method to apply text-mining to EHRs for the identification of patient characteristics. The secondary outcomes included the performance of text-mining against human intelligence measured in recall, specificity, precision, negative predictive value (NPV) and the F1-score. Descriptive data analysis was used to analyze the model performance using R (version 4.4.1) in combination with RStudio. Modelling and evaluation were performed in Jupyter Notebooks running on Python 3.8.

## Results

In this study, we analyzed 1,120 readmission files as shown in Table 1. Among these, 221 readmissions involved patients identified with a language barrier. In 328 readmissions, patients were identified as living alone, in 185 readmissions patients were cognitive frail and in 24 readmissions, the patient was non-adherent.

**Table 1:** Characteristics of the study population (*n*=1,120 readmissions for 878 patients)

	Total population
Characteristics	*n* = 1,120 readmissions
Male, n (%)	556 (49.6)
Age in years, mean (SD)	64 (17)
Department, n (%)	
Cardiology	147 (13.1)
Surgery	286 (25.5)
Internal	260 (23.2)
Pulmonology	226 (20.2)
Gastroenterology	144 (12.9)
Neurology	45 (4.0)
Psychiatry	12 (1.1)
Patient characteristics	
Language barrier, n (%)	221 (19.7)
Living alone, n (%)	328 (29.3)
Conflicting information ^a^	35 (3.1)
Cognitive frailty, n (%)	185 (16.5)
Conflicting information ^a^	7 (0.6)
Suspicion of non-adherence, n (%)	24 (2.1)

^a^ Conflicting information refers to EHRs containing contradicting information making a final classification unobtainable

The first step was to evaluate the complexity of the patient characteristic terms in order to assess whether RB queries or NER models would be most suitable for the term. To distinguish between terminology suitable for RB queries versus NER models, we categorized patient characteristics as having either ‘simple’ or ‘complex’ terminology. This classification was based on a combination of factors: the number of unique expressions found in the annotated data, the degree of linguistic variation, and the potential for semantic ambiguity. Characteristics with fewer than 30 distinct expressions and low variability in phrasing —such as language barrier—were considered to have ‘simple’ terminology. In contrast, the characteristics living situation, cognitive frailty and non-adherence exhibited more linguistic diversity and context-dependent meaning, qualifying as ‘complex’.

Table 2 showcases the in- and exclusion criteria for the language barrier RB query based on the extracted text from the EHRs (*for annotation lists of the other patient characteristics refer to the appendix*). The final performance results of the text-mining models are presented in Table 3* (for full model specifics*,* refer to the appendix).*

**Table 2:** In- and exclusion criteria for the RB query language barrier (translated to English)

Inclusion terms	Exclusion terms
language disorder language barrier language, barrier language, barriere language, barier doesn’t speak Dutch	language, gestures language, proficient language, Dutch language, undisturbed speech, undisturbed language, undisturbed normal, language normal, speech speech, no dysarthia language, no dysarthia speaks, no Dutch none, language disorder none, speech disorder none, speech or language disorder language, normal speech, normal speech and language”, normal language, intact speech, intact speech and language intact language, no deviations speech, no deviations speech and language no deviations language, good language disorder, stroke speaks, sentences speaks, “full sentences

**Table 3:** Performance indicators of the text-mining models used to identify four patient characteristics from EHRs

Patient characteristics	Recall (sensitivity)	Specificity	Precision (PPV)	NPV	F1-score
	Performance based on train/test set				
*Language barrier*	0.99	0.96	0.87	1.00	0.93
*Living alone*	0.86	0.94	0.85	0.94	0.85
*Cognitive frailty*	0.59	0.76	0.95	0.20	0.73
*Non-adherence*	0.75	0.99	0.56	0.99	0.64
	Performance based on internal validation dataset				
*Living alone*	0.81	1.00	1.00	0.84	0.90
*Cognitive frailty*	0.73	0.96	0.95	0.78	0.83
*Non-adherence*	0.90	0.99	0.99	0.91	0.95

The RB query for language barrier showed robust performance across all metrics, with a recall of 0.99 and specificity of 0.96. Table 4 provides insight into factors that led to false positive and false negative classifications for each model. This outlines that while the model was effective in identifying language barriers, the model did miss certain specific terms. As the RB query lacks any machine learning or predictive functionality, evaluation using an internal validation set was not feasible.

The NER model for living alone showed a recall of 0.86 and specificity of 0.94. Here, the model was good at excluding patients that do not live alone but seemed to misclassify ‘independent’ as living alone in some cases. The internal validation dataset showed a recall of 0.81 and specificity of 1.00, indicating good performance.

The NER model for cognitive frailty showed a recall of 0.59 and specificity of 0.76, accurately recognizing terms like ‘forgetful’ and ‘cognitive impairment’. However, it overlooked terms such as dementia and Alzheimer’s disease as these were not often present in the dataset (Table 4), possibly relating to the relatively low NPV (0.20). The internal validation dataset showed higher performance metrics with a recall of 0.73 and specificity of 0.96.

The NER model for non-adherence demonstrated a recall of 0.75 and a specificity of 0.99. As shown in Table 4, it struggled with intentional starting/stopping medication, resulting in a relatively low PPV (0.56). Performance of the internal validation set showed a recall of 0.90 and specificity of 0.99.

Overall, the specificity and NPV for all models, except cognitive frailty, were relatively high, indicating their proficiency in correctly excluding patients without the patient characteristics. Performance of the models on the internal validation sets seemed to perform slightly better than the train set.

**Table 4:** An overview of the strong and poor performance aspects of the text-mining models

Model	Strong performance	False positive classification	False negative classification
Language barrier (RB)	Good identification of almost all patients with a language barrier (true positives)	Difficulty differentiating between language barriers related to family / friends instead of patient e.g. *“Mother of patient had language barrier”*	Missed patients with more nonspecific terms for language barrier e.g. “*patient doesn’t understand what I say”*
Living alone (NER)	Good at excluding patients in institutions; Good recognition of widowed patients;	False positive classification of patients due to ambiguity of terms like “independently” e.g. *“lives independently with less vital wife and 2 granddaughters”;* Patients that were home alone at the time a symptom occurred, but do not truly live alone e.g. “*was home alone*”;	Difficulty when time influences discharge location, such as discussions if the patient should go to institution / receive home care / temporary caregivers because they live by themselves e.g. “*I think patient might need to go to institution after discharge as she can’t move independently”*
Cognitive frailty (NER)	Good identification of descriptions containing “cognitive” such as cognitive decline, cognitive impairment, cognitive disorders; Good identification of notes containing “forgetful”	False positive classification of clinical notes where practitioner questions whether there is forgetfulness; “*Patient seems a bit confused but I think this is due to the language barrier*” Misclassification when healthcare professionals forgot something, e.g. “*I forgot to measure patient’s temperature yesterday*”	Poor identification of delirium; Poor identification of Alzheimer; Difficulty classifying short term memory loss; Difficulty identifying patients with nonspecific terms for forgetful e.g. *“patient is in search of words”*
Non-adherence (NER)	Good at identifying patients that are truly non-adherent: e.g. “*patient refuses to take medication*” or e.g. “*patient stopped taking medication a while ago*”	False inclusion of clinical notes referring to intentionally starting/stopping medication e.g. *“Stopped the antibiotics because patient recovered”* False inclusion of text unrelated to non-adherence e.g. “*no medication was administered before this intake*”	Unable to accurately extract information for specific medicine e.g. *“used to take lamotrigine but stopped because she was getting palpitations”*

## Discussion

In assessing our primary aim, we devised a feasible method for the application of text-mining to identify patient characteristics from EHRs. The models attained an average recall of 0.83 and a precision average of 0.89. The RB query for language barrier and the NER model for living alone emerged as overall top performers. The primary challenges affecting performance, particularly the PPV, were term ambiguity and varying interpretations of the same word, which led to misclassifications.

Previous research corroborates the viability of text-mining in analyzing extensive patient files and the effectiveness of RB queries for clear-cut variables [12, 24]. It also seems RB queries cannot be optimized when patient files contain abbreviations and spelling mistakes, as the query will not be able to define them all [5, 25]. Additionally, our findings align with existing literature indicating the time efficiency of NER models over RB queries, corroborated by our experience of an extensively revised annotation list facilitating the NER model development [18]. 

Interestingly, our research contrasts with prior studies where NER models typically outperform RB queries [18]. The best-performing model in our study was the RB query for language barrier, followed by the NER model for living alone. This suggests that NER models may show better performance over RB queries when context understanding is crucial. Although NER models adeptly identified patient characteristics, they struggled with terms having multiple meanings. Additionally, the ‘black box’ nature of NER models poses challenges in result interpretation, necessitating a thorough understanding of the input data. Enhancing understanding could involve displaying texts used by the models for positive classifications of specific variables [13, 26]. 

The RB query for language barrier incorrectly classified language barriers of family members to the patient, leading to a slightly lower PPV. The NER model for living alone mistakenly identified patients living ‘independently’ as living alone, whilst this could also refer to geriatric patients living at home without homecare, leading to a slightly lower PPV. Here, it seems interpretation of the context is key for identifying the right patients. The NER model for cognitive frailty scored slightly lower for recall and low for NPV. This limits the model’s utility in excluding patients without cognitive frailty. This poor performance is likely a result of strong class imbalance, as cognitive frailty was relatively rare in the dataset. As a result, the model produced a substantial number of false negatives. Although precision and F1-score were acceptable, the low NPV highlights that this model is not suitable for use as a standalone screening tool. Noticeably, the performance of the model showed stronger results on the internal validation set. This could possibly be due to the validation set containing terminology that was similar to terminology that the model was trained on. This phenomenon, referred to as underlying influence, is addressed in the limitations. Lastly, the NER model for non-adherence mistakenly classified intentional starting / stopping of medication as non-adherent. It seems that because non-adherence was often described by the healthcare practitioner in relation to specific drugs, the model had more difficulty identifying these patients as it was not trained specifically with all drug names, leading to slightly lower performance metrics.

We hypothesize that with further refinement, these models could be integrated into hospital software, providing personalized care. An important consideration in interpreting our findings is the language-specific nature of clinical NER. The models were developed using Dutch clinical notes, and to our knowledge, few to no pre-trained Dutch clinical NER models currently exist. As a result, all annotation and model training had to be developed from scratch. While the methodology—including annotation design, model pipeline, and evaluation—can be applied in other languages or clinical contexts, the linguistic characteristics (e.g., grammar, terminology, phrasing) and documentation habits may vary across healthcare systems. Therefore, transferring these models directly to other languages without linguistic adaptation may result in reduced performance. Nonetheless, the structured approach is transferable and can serve as a template for NER development in other languages with appropriate domain-specific tuning.

Early identification of patient characteristics such as language barriers or cognitive frailty can enable healthcare professionals to take preemptive measures, such as arranging caregiver presence during appointments and discharge education on medication. Enhanced monitoring for patients living alone or with language barriers post-discharge could also mitigate adverse events. Such proactive approaches could significantly improve communication, follow-up care, medication adherence, and ultimately lead to improved clinical outcomes, including a reduction of unplanned hospital readmissions [27]. 

### Strengths

A primary strength of this study is its pioneering analysis of the four specified patient characteristics from patient files, with the developed models demonstrating promising outcomes. Additional strengths include the utilization of a substantial manual cohort (1,120 readmission files) and the implementation of descriptive variation analysis to pre-determine the most fitting text-mining technique. Furthermore, the research benefitted from the insights of an interdisciplinary expert team, including (clinical) pharmacists, a neurologist, and data scientists. This collaboration enriched the study with both practical clinical perspectives and technical expertise, facilitating the effective and straightforward application of these complex models in a clinical environment. Lastly, the performance on the internal validation sets proves these models could operate in a real-life setting.

### Limitations

However, the study is not without its limitations. Primarily, the models were developed based on a manual standard, which, being subject to human error, could lead to misclassifications. Instances where the model’s outputs disagreed with the manual standard were generally interpreted as model errors [28]. To counter potential misclassifications in the manual standard, we employed a thorough process, extracting exact text for each variable through a joint review. A limitation of this approach, however, is that inter-annotator agreement (IAA) metrics were not calculated since classifications were refined using a consensus process—without independent parallel annotations. Therefore, standard IAA metrics such as Cohen’s Kappa could not be applied. This limits the ability to assess the potential upper bound of model performance.

Another limitation of this study is that the training dataset was selected through random sampling without stratification by patient characteristics. As a result, certain characteristics may have been underrepresented in the training data. However, this approach was chosen to reflect the natural variability and documentation practices within clinical notes, where relevant characteristics are often recorded across a wide range of patient types. Additionally, temporal ambiguity presents a particular challenge for specific patient characteristics. Although we used the most recent clinical notes, these may still contain historical information (e.g., “the patient had previously shown signs of cognitive frailty”), partly due to copy-paste practices from historical clinical notes. To reduce this risk, we focused on relevant note types and excluded explicit historical indicators (e.g., “patient history”), but some temporal ambiguity may remain.

Although the training and test datasets were randomly split from the full dataset to prevent selection bias, we acknowledge the potential presence of underlying influences in the data. Specifically, different clinical notes written by the same healthcare professionals may appear in both the training and test sets. As clinicians tend to use consistent language patterns and terminology, this may inadvertently introduce similarities across datasets, leading to information leakage. This phenomenon, which we refer to as underlying influence, reflects real-world practice variation in clinical documentation and is inherent to the use of narrative EHR data. While controlling for this (e.g., by splitting data by author or department) could reduce this effect, such an approach was not feasible in this study due to the limited number of contributing clinicians and the need to preserve sufficient data for training. Future research should explore stratified sampling or author-based splitting to further mitigate this influence. The absence of a systematic error analysis also limits our ability to precisely identify the linguistic patterns that contribute to misclassifications, which is critical for refining model logic and ensuring safe deployment in clinical workflows. Future research should incorporate tools such as ELI5 or SHAP to support transparent model refinement and inform targeted post-processing rules.

## Conclusion

This study has successfully demonstrated the feasibility of using text-mining to extract patient characteristics from EHRs. The models show high recall, underscoring that the selection between an RB query and a NER model depends on term interpretation and ambiguity. These findings have potential implications for identifying patient characteristics, in this case specifically language barrier, living alone, cognitive frailty, and non-adherence, which are crucial in classifying vulnerable patients. This supports the significance of incorporating patient characteristics in patient care.

In future work, we aim to further refine these models to enhance healthcare decision-making and reduce administrative burdens. This continual improvement aligns with the evolving nature of healthcare and the increasing relevance of text-mining in clinical settings.

## Supplementary Information

Below is the link to the electronic supplementary material.

Supplementary Material 1

## Data Availability

Data is provided within the manuscript or supplementary information files. The datasets generated and analysed during the current study are not publicly available due privacy restrictions but are available from the corresponding author on reasonable request.
