# Supplementary material for: Applying text-mining to clinical notes: the identification of patient characteristics from electronic health records (EHRs)
Source: BMC Med Inform Decis Mak. 2025 Aug 12;25:302. doi: 10.1186/s12911-025-03137-x (PMC12344823; doi:10.1186/s12911-025-03137-x)
Supplement: Supplementary file 1 — Supplementary Material 1 [file 12911_2025_3137_MOESM1_ESM.docx]

**Appendix**

A.1 is the RB query used in this study. The original Dutch queries were translated using Google translate. The English query was then reviewed for its completeness and resemblance to the Dutch query. A.2 – A.4 are the annotation lists used for the NER models.

A.1 Language barrier: RB query

| **Dutch query** | **English query** |
| --- | --- |
| **--Inclusion**  and (  (CONTAINS(notetext.Text, 'taalstoornis'))  OR (CONTAINS(notetext.Text, 'taalbarriere'))  OR (CONTAINS(notetext.Text, 'NEAR((taal, barriere), 5)'))  OR (CONTAINS(notetext.Text, 'NEAR((taal, barierre), 5)'))  OR (CONTAINS(notetext.Text, 'NEAR((taal, barrière), 5)'))  OR (CONTAINS(notetext.Text, 'NEAR(("spreekt geen", Nederlands), 2)'))) | **--Inclusion**  and (  (CONTAINS(notetext.Text, 'language disorder'))  OR (CONTAINS(notetext.Text, 'language barrier'))  OR (CONTAINS(notetext.Text, 'NEAR((language, barrier), 5)'))  OR (CONTAINS(notetext.Text, 'NEAR((language, barriere), 5)'))  OR (CONTAINS(notetext.Text, 'NEAR((language, barier), 5)'))  OR (CONTAINS(notetext.Text, 'NEAR(("doesn't speak", Dutch), 2)'))) |
| **Exclusion** | **Exclusion** |
| and (  (NOT CONTAINS(notetext.Text, 'NEAR((taal, gebaren), 5)'))  AND (NOT CONTAINS(notetext.Text, 'NEAR((taal, vaardig), 5)'))  AND (NOT CONTAINS(notetext.Text, 'NEAR((taal, Nederlands), 5)'))  AND (NOT CONTAINS(notetext.Text, 'NEAR((taal, ongestoord), 5)'))  AND (NOT CONTAINS(notetext.Text, 'NEAR((spraak, ongestoord), 5)'))  AND (NOT CONTAINS(notetext.Text, 'NEAR((spraak, "niet gestoord"), 5)'))  AND (NOT CONTAINS(notetext.Text, 'NEAR((taal, "niet gestoord"), 5)'))  AND (NOT CONTAINS(notetext.Text, 'NEAR((normale, taal), 5)'))  AND (NOT CONTAINS(notetext.Text, 'NEAR((normale, spraak), 5)'))  AND (NOT CONTAINS(notetext.Text, 'NEAR((spraak, "geen dysartie"), 5)'))  AND (NOT CONTAINS(notetext.Text, 'NEAR((taal, "geen dysartie"), 5)'))  AND (NOT CONTAINS(notetext.Text, 'NEAR((spreekt, "geen Nederlands"), 5)'))  AND (NOT CONTAINS(notetext.Text, 'NEAR((geen, taalstoornis), 2)'))  AND (NOT CONTAINS(notetext.Text, 'NEAR((geen, spraakstoornis), 2)'))  AND (NOT CONTAINS(notetext.Text, 'NEAR((geen, "spraak of taalstoornis"), 1)'))  AND (NOT CONTAINS(notetext.Text, 'NEAR((taal, normaal), 1)'))  AND (NOT CONTAINS(notetext.Text, 'NEAR((spraak, normaal), 1)'))  AND (NOT CONTAINS(notetext.Text, 'NEAR(("spraak en taal", normaal), 1)'))  AND (NOT CONTAINS(notetext.Text, 'NEAR((taal, intact), 1)'))  AND (NOT CONTAINS(notetext.Text, 'NEAR((spraak, intact), 1)'))  AND (NOT CONTAINS(notetext.Text, 'NEAR(("spraak en taal", intact), 1)'))  AND (NOT CONTAINS(notetext.Text, 'NEAR((taal, "geen afwijkingen"), 1)'))  AND (NOT CONTAINS(notetext.Text, 'NEAR((spraak, "geen afwijkingen"), 1)'))  AND (NOT CONTAINS(notetext.Text, 'NEAR(("spraak en taal", "geen afwijkingen"), 1)'))  AND (NOT CONTAINS(notetext.Text, 'NEAR((taal, gb), 1)'))  AND (NOT CONTAINS(notetext.Text, 'NEAR((taalstoornis, stroke), 5)'))  AND (NOT CONTAINS(notetext.Text, 'NEAR((spreekt, volzinnen), 2)'))  AND (NOT CONTAINS(notetext.Text, 'NEAR((spreekt, "volle zinnen"), 2)')) | and (  (NOT CONTAINS(notetext.Text, 'NEAR((language, gestures), 5)'))  AND (NOT CONTAINS(notetext.Text, 'NEAR((language, proficient), 5)'))  AND (NOT CONTAINS(notetext.Text, 'NEAR((language, Dutch), 5)'))  AND (NOT CONTAINS(notetext.Text, 'NEAR((language, undisturbed), 5)'))  AND (NOT CONTAINS(notetext.Text, 'NEAR((speech, undisturbed), 5)'))  AND (NOT CONTAINS(notetext.Text, 'NEAR((language, "undisturbed"), 5)'))  AND (NOT CONTAINS(notetext.Text, 'NEAR((normal, language), 5)'))  AND (NOT CONTAINS(notetext.Text, 'NEAR((normal, speech), 5)'))  AND (NOT CONTAINS(notetext.Text, 'NEAR((speech, "no dysarthia"), 5)'))  AND (NOT CONTAINS(notetext.Text, 'NEAR((language, "no dysarthia"), 5)'))  AND (NOT CONTAINS(notetext.Text, 'NEAR((speaks, "no Dutch"), 5)'))  AND (NOT CONTAINS(notetext.Text, 'NEAR((none, language disorder), 2)'))  AND (NOT CONTAINS(notetext.Text, 'NEAR((none, speech disorder), 2)'))  AND (NOT CONTAINS(notetext.Text, 'NEAR((none, "speech or language disorder"), 1)'))  AND (NOT CONTAINS(notetext.Text, 'NEAR((language, normal), 1)'))  AND (NOT CONTAINS(notetext.Text, 'NEAR((speech, normal), 1)'))  AND (NOT CONTAINS(notetext.Text, 'NEAR(("speech and language", normal), 1)'))  AND (NOT CONTAINS(notetext.Text, 'NEAR((language, intact), 1)'))  AND (NOT CONTAINS(notetext.Text, 'NEAR((speech, intact), 1)'))  AND (NOT CONTAINS(notetext.Text, 'NEAR(("speech and language", intact), 1)'))  AND (NOT CONTAINS(notetext.Text, 'NEAR((language, "no deviations"), 1)'))  AND (NOT CONTAINS(notetext.Text, 'NEAR((speech, "no deviations"), 1)'))  AND (NOT CONTAINS(notetext.Text, 'NEAR(("speech and language", "no deviations"), 1)'))  AND (NOT CONTAINS(notetext.Text, 'NEAR((language, good), 1)'))  AND (NOT CONTAINS(notetext.Text, 'NEAR((language disorder, stroke), 5)'))  AND (NOT CONTAINS(notetext.Text, 'NEAR((speaks, sentences), 2)'))  AND (NOT CONTAINS(notetext.Text, 'NEAR((speaks, "full sentences"), 2)')) |

A.2 Living alone: NER model annotation list

| **Dutch** | **English** |
| --- | --- |
| Woont alleen  Alleenwonend  Alleen wonen  Woont zelfstandig  Alleen thuis  Heeft niemand thuis  Is weduwe  Is weduwnaar  Is alleenstaand  Partner overleden  Woont op zichzelf | Lives alone  Living alone  Live on your own  Lives independently  Home alone  Doesn't have anyone home  Is widow  Is a widower  Is single  Spouse passed away  Lives on his own  Lives on her own |

A.3 Cognitive frailty: NER model annotation list

| **Dutch** | **English** |
| --- | --- |
| Cognitieve achterstand  Cognitief functioneren  Delier  Alzheimer  Vergeetachtig  Vergeet  Verward  Dysfagie  Geheugenproblemen  Korte termijn geheugen  Geheugen stoornis  Cognitief beperkt  Cognitieve achteruitgang  Cognitieve ontwikkelingsachterstand  Dementie  Korsakoff  Mentale retardatie  Verwardheid | Cognitive Retardation  Cognitive functioning  Delirium  Alzheimer  Forgetful  Forget  Confused  Dysphagia  Memory problems  Short-term memory  Memory disorder  Cognitive limited  Cognitive decline  Cognitive developmental delay  Dementia  Korsakoff  Mental retardation  Confusion |

A.4 Therapy adherence: NER model annotation list

| **Dutch** | **English** |
| --- | --- |
| Zelf gestopt  Bijwerkingen  Vergeten in te nemen  Therapieontrouw  Niet ingenomen  Medicatie ontrouw  Niet medicatie trouw  Ontrouw | Stopped by themself  Side effects  Forgot to take  Therapy non-adherence  Therapy non-compliance  Medication non-adherence  Medication non-compliance  Non-compliant  Not taken medication |
